# Sepsis with *Enterococcus* and *Klebsiella* in a patient with recent invasive dental procedures, endocarditis and subsequent intestinal abscess

**DOI:** 10.1186/1471-2334-13-S1-P35

**Published:** 2013-12-16

**Authors:** Anca Streinu-Cercel, Oana Streinu-Cercel, Alina Cristina Neguț, Maria Magdalena Moțoi, Mihai Săndulescu, Luminița Bradu, Ioana Berciu, Adrian Streinu-Cercel

**Affiliations:** 1 Carol Davila University of Medicine and Pharmacy, Bucharest, Romania; 2 National Institute for Infectious Diseases “Prof. Dr. Matei Balş”, Bucharest, Romania

## Background

Fever is one of the most suggestive symptoms for infectious disease pathology, although it is neither specific nor sensitive. Reoccurrence of fever during the course of hospital admission generally suggests that the diagnosed infection might not be controlled and that medical management should be revised. However, other concurrent illnesses can also be responsible for fever; for this reason, a complete interdisciplinary medical investigation should be performed.

## Case report

We present the case of a 48 year-old patient, smoker, moderate alcohol consumer, with grade 2 obesity, arterial hypertension, COPD and hepatic steatosis.

He was admitted to the intensive care unit of our Infectious Disease Clinic for fever and chills; blood cultures positive for *Enterococcus* and *Klebsiella* XDR. A diagnosis of endocarditis and sepsis with multiple organ dysfunction syndrome was established. He received treatment with meropenem, colistin, linezolid, followed by tigecycline.

The patient’s medical history is positive for an invasive procedure the day before fever onset: post-extractional ridge preservation with bone substitute of bovine origin. After consult with the dental surgeon, the alveolar addition material was extracted and sent to the laboratory for culture – results came out positive for *Enterococcus* spp and *Klebsiella* spp, antibiotic therapy was continued and the patient presented favorable clinical evolution.

Transesophageal echocardiography revealed a hyperechoic immobile image of 3/4 mm on the aortic left coronary cusp, compatible with an old vegetation.

Over the course of hospitalization, the patient developed abdominal pain, and fever reappeared. Abdominal CT scans were suggestive of hydropneumoperitoneum through possible digestive perforation and the patient was transferred to a surgery department where he was diagnosed with parieto-sigmoid and intermesenterico-sigmoido-jejunal abscess due to infection of sigmoid diverticula. After abscess drainage and lavage, fever persisted, and he was transferred to our Clinic, where antibiotic therapy was continued and the patient presented favorable clinical evolution.

## Conclusion

Fever of recent onset in a patient with existing, but undiagnosed comorbidities can be tricky to manage. It is important to try to differentiate the etiology of recurrent feverish episodes. Although the simplest explanation is oftentimes the best, different concurrent pathologies can sometimes justify the same sign or symptom, as was the case with this patient, where the second course of fever did not signal an issue in the management of endocarditis, but rather the development of a complication of a pre-existing pathology that had not been yet diagnosed: sigmoid diverticula.

